# Association of Antihistamine Use with Increased Risk of Esophageal Squamous Cell Carcinoma: A Nationwide, Long-Term Follow-Up Study Using Propensity Score Matching

**DOI:** 10.3390/biomedicines11020578

**Published:** 2023-02-16

**Authors:** Jhao-Yang Peng, Ying-Hui Yu, Wan-Ming Chen, Ben-Chang Shia, Mingchih Chen, Szu-Yuan Wu

**Affiliations:** 1 Graduate Institute of Business Administration, College of Management, Fu Jen Catholic University, Taipei 242062, Taiwan; 2 Department of Colorectal Surgery, Lo-Hsu Medical Foundation, Lotung Poh-Ai Hospital, Yilan 265, Taiwan; 3 Artificial Intelligence Development Center, Fu Jen Catholic University, Taipei 242062, Taiwan; 4 Department of Food Nutrition and Health Biotechnology, College of Medical and Health Science, Asia University, Taichung 413, Taiwan; 5 Division of Radiation Oncology, Lo-Hsu Medical Foundation, Lotung Poh-Ai Hospital, Yilan 265, Taiwan; 6 Big Data Center, Lo-Hsu Medical Foundation, Lotung Poh-Ai Hospital, Yilan 265, Taiwan; 7 Department of Healthcare Administration, College of Medical and Health Science, Asia University, Taichung 413, Taiwan; 8 Cancer Center, Lo-Hsu Medical Foundation, Lotung Poh-Ai Hospital, Yilan 265, Taiwan; 9 Centers for Regional Anesthesia and Pain Medicine, Taipei Municipal Wan Fang Hospital, Taipei Medical University, Taipei 110, Taiwan; 10 Department of Management, College of Management, Fo Guang University, Yilan 265, Taiwan

**Keywords:** H1-antihistamine, esophageal squamous cell carcinoma, incidence rate, hazard ratio, dose–response relationship

## Abstract

Esophageal cancer is a common and aggressive cancer, with a five-year survival rate of approximately 20%. Therefore, identifying safe and effective medications that can reduce the risk of esophageal cancer is of great importance. Objective: To examine the association between H1-antihistamines (AHs) use and the incidence of esophageal squamous cell carcinoma (ESCC) in a head-to-head propensity score matching (PSM) comparative study. Design: Retrospective cohort study. Setting: Nationwide population-based study in Taiwan. Participants: 1289,526 adults from the National Health Insurance Research Database from 2008 to 2018. Exposures: AH use. Main Outcomes and Measures: Incidence rates (IRs), incidence rate ratios (IRRs), and adjusted hazard ratios (aHRs) of ESCC in AH users compared with nonusers. Results: AH users had a significantly higher IR of ESCC than nonusers (1.47 vs. 1.36 per 100,000 person-years). The IRR (95% CI) for ESCC was 1.18 (1.08–1.28) in AH users compared with nonusers. After adjustment for age, sex, income levels, urbanization, cigarettes smoking, alcoholic related diseases, comorbidities, medication use, and Charlson Comorbidity Index scores, the aHR (95% CI) for ESCC was 1.22 (1.12–1.33) in AH users compared with nonusers. A dose–response relationship was also observed, with aHRs for AH use at 28–182, 183–488, 489–1043, and >1043 cumulative defined daily doses (cDDDs) of 1.12, 1.20, 1.25, and 1.37, respectively, compared with <28 cDDDs. Conclusions and Relevance: Our study found a significant association between AH use and the increased risk of ESCC, with a dose–response relationship. This study suggests that AH use may increase the risk of ESCC, especially at high doses, and highlights the importance of caution when prescribing AHs.

## 1. Introduction

Esophageal cancer is a major global health concern, ranking as the eighth most common cancer and the sixth most common cause of death worldwide [1,2,3]. Squamous cell cancer has historically made up the majority of esophageal cancers globally [1,2,3]. In Taiwan, esophageal cancer is a leading cause of cancer-related deaths, with over 95% of cases classified as esophageal squamous cell carcinoma (ESCC) [4,5,6]. The pathological types of ESCC in Taiwan and other Asian countries tend to differ from those in Western countries, where esophageal adenocarcinoma is more common [7,8]. In the past two decades, treatment options for ESCC have been limited to surgery, chemotherapy with platinum-based regimens, and radiotherapy, with little improvement in outcomes [4,5,6,9,10]. The overall survival rate for ESCC is poor both globally and in Taiwan [4,5,6,10]. The mean age of individuals diagnosed with ESCC in Taiwan is around 50 years old, and these patients are often the main economic supports for their families and countries [4,5,6,10]. Therefore, finding effective protective medications for ESCC is a valuable and important goal for the general population.

Antihistamines targeting the histamine receptor H1 are promising candidates for repurposing as cancer therapies due to their safety, minimal side effects, and widespread tolerance among patients [11]. There is growing evidence that they may be effective against tumors [12,13,14,15,16,17,18,19,20]. Preclinical studies have shown that H1-antihistamines, such as cyproheptadine, can suppress tumorigenesis and inhibit tumor growth [19,21,22,23]. Preclinical and clinical studies have also revealed an association between H1-antihistamine use, particularly cyproheptadine, and increased survival in patients with hepatocellular carcinoma (HCC) [14,15,21,22,23,24,25,26,27,28]. This association also extends to a protective effect against HCC risk in individuals infected with hepatitis B or C virus, and an enhancement of cancer cell death in HCC [14,15,21,22,23,24,25,26,27,28]. In addition, two separate studies have suggested that H1-antihistamine use, regardless of the specific type, may reduce the risk of HCC in type 2 diabetes patients, as well as in individuals with hepatitis B or C [13,15]. The potential protective effects of AHs against cancer risk may include the alleviation of allergic reactions, activation of mitogen-activated protein kinases, inhibition of the combination of autophagosomes and lysosomes, anti-inflammation, and immunoregulation [12,13,14,15,16,17,18,19,20,21,22,23]. However, until now, there have been no reports on the association between AH use and risk of ESCC. Despite their long-term use and safety as traditional medications, further research is needed to determine whether AHs may be a viable option for the prevention of ESCC.

To better understand the association between AH use and the risk of ESCC, as well as the potential dose–response relationship, we conducted a long-term follow-up head-to-head propensity score matching (PSM) comparative national cohort study. This is the first clinical study of its kind to investigate the relationship between AH use and ESCC risk, and it provides valuable information that can be used to guide future research on ESCC risk.

## 2. Methods

### 2.1. Study Population

We conducted a population-based cohort study using data from the Taiwan National Health Insurance (NHI) Research Database (NHIRD) from 2008 to 2020. The NHIRD contains comprehensive medical claims data for all NHI beneficiaries, including diagnoses, procedures, drug prescriptions, demographics, and enrollment profiles, all of which are encrypted using unique patient identifiers [13,29,30,31,32,33]. The NHIRD is linked to the death registry, allowing us to determine the vital status and cause of death for each included patient. The NHIRD is a valuable resource for population-based research, as it covers the entire NHI-insured population of Taiwan, which represents over 99% of the Taiwanese population [13,30,31,32,33].

Our study included patients aged 40 or older who were enrolled in the NHIRD and excluded patients with missing age data. AH use was defined as the use of at least 28 cumulative defined daily doses (cDDDs) of an AH. The index date was the date when a patient began using at least 28 cDDDs of an AH. The observation period for each patient began on the index date and continued until the patient was diagnosed with ESCC, died, or the end of the study period (31 December 2021), whichever occurred first. Patients who were prescribed at least 28 cDDDs of an AH during the follow-up period formed the case group (AH users), while those who were prescribed less than 28 cDDDs of an AH during the follow-up period formed the control group (AH nonusers). The follow-up duration was defined as one year after the initial AH use or cohort entry date. This study is the first to investigate the association between AH use and ESCC risk, and aims to provide valuable information for understanding the risk of ESCC in the general population.

We excluded patients from our cohort who met any of the following criteria: (1) they were diagnosed with ESCC within 1 year of the index date, (2) they had missing data on their sex or age or were younger than 40 years old, (3) they had a follow-up duration of less than 1 year, or (4) they were diagnosed with any other type of cancer within 1 year before the cohort entry date (to prevent the influence of ESCC-related metastases). This was carried out to ensure that our results accurately reflected the association between AH use and ESCC risk.

The study protocols were reviewed and approved by the Institutional Review Board of Tzu-Chi Medical Foundation (IRB number: IRB109-015-B).

### 2.2. Study Covariates

To control for potential confounding factors, we included various covariates in our analysis. The study participants were divided into four age groups based on their age at the index date: 40–50, 51–60, 61–70, and 71 or older. The index date for AH users was defined as the date when they started using AHs at a dose of at least 28 cDDDs. For matched AH nonusers, we used variables collected at the index date. To prevent repeated adjustment in multivariate analysis, we excluded repeat comorbidities from the CCI calculations. We identified comorbidity onset within one year before the index date using International Classification of Diseases codes from either the main inpatient diagnosis or at least two outpatient visits within one year. These codes were from either the Ninth Revision, Clinical Modification (ICD-9-CM) or the Tenth Revision, Clinical Modification (ICD-10-CM).

### 2.3. AH Exposure

AH use was defined as the use of at least 28 cDDDs of an AH [13,15]. AHs were prescribed for the treatment of symptoms related to asthma, allergic rhinitis, medication allergies, environmental allergies, or viral infections (such as runny nose, itchy eyes, and pruritus). Information on drug type, dosage, administration route, prescription date, and total number of pills dispensed by the pharmacy was collected. Because AH use may have occurred in separate years during the study period and patients may have changed their drug use patterns over time, we treated AH use as a time-varying covariate in the Cox model. The cumulative dose of AHs was calculated by multiplying the number of pills dispensed by the prescribed dose and then dividing the result by the recorded days’ supply. The defined daily dose (DDD) of AHs, as established by the World Health Organization, was used to express dosage. The DDD is the average maintenance dose per day for a drug used for its main indication in adults. The cDDDs were calculated as the sum of the daily defined doses. AH nonuse was defined as less than 28 cDDDs to exclude occasional AH use, while AH use was defined as at least 28 cDDDs. All patients were divided into four subgroups based on quartiles of cDDDs (Table 1).

To further investigate the potential relationship between AH use and ESCC risk, we conducted a sensitivity analysis to examine the intensity of AH use. We calculated the average daily dose of AHs by dividing the DDD by the total number of prescription days. AH use intensity was then divided into two categories: average DDDs of >1 or ≤1. This allowed us to evaluate the frequency of daily AH use and its potential effect on ESCC risk.

### 2.4. PSM and Covariates

We used a time-varying Cox proportional hazards model to analyze the relationship between AH use and the onset of ESCC after controlling for potential confounders. To minimize the impact of confounding factors when comparing the risk of ESCC in AH users and nonusers, we matched the patients based on their propensity scores. The variables used for matching included age, sex, income level, urbanization, cigarette smoking, alcoholic related diseases, and comorbidities such as diabetes, hypertension, hyperlipidemia, chronic obstructive pulmonary disease, gastroesophageal reflux disease, Barrett’s esophagus, obesity, achalasia, Tylosis (Howel–Evans syndrome), Plummer–Vinson syndrome, and medication use (aspirin, metformin, statin, proton pump inhibitor [PPIs]). Comorbidities were identified using the International Classification of Diseases, Ninth Revision, Clinical Modification or International Classification of Diseases, Tenth Revision, Clinical Modification codes from one inpatient visit or two or more outpatient visits within 1 year for the main diagnosis. We excluded repeat comorbidities from the CCI calculations to prevent repetitive adjustment in the multivariate analysis.

Here, the continuous variables are presented as means with standard deviations or medians with first and third quartiles, as appropriate. To minimize differences between the patient groups, we used a matching technique called greedy method: PSM with a caliper width of 0.2 to match the patients at a ratio of 1:1 [34]. This method involves selecting controls with identical background covariates that the investigator deems necessary to control for.

### 2.5. Primary Endpoints

The primary outcome of this study was the occurrence of ESCC, which was confirmed through the certification record in the Registry for Catastrophic Illness Patients [35].

### 2.6. Sensitivity Analysis

To further explore the relationship between AH use and ESCC risk, we conducted sensitivity analyses. We examined the risk of ESCC in patients with different levels of AH use intensity by dividing the patients into two subgroups based on the average daily DDD: >1 or ≤1. This allowed us to evaluate the effect of the frequency of daily AH use on ESCC risk. We also conducted stratified analyses by age, sex, cigarette smoking, alcohol-related diseases, and medication use (aspirin, metformin, statin, PPIs) to assess the potential effect modification of these factors on the association between AH use and ESCC risk.

### 2.7. Statistical Analysis

We collected information on patient characteristics including age, sex, comorbidities, and AH dosage. Age was divided into 10-year intervals and the baseline characteristics of AH users and nonusers were compared using chi-squared test for categorical variables, t-test for continuous variables, and Wilcoxon rank-sum test for median values. The cohort entry date was set as the baseline. To assess the association between AH use and risk of ESCC, we calculated incidence rates (IRs) and incidence rate ratios (IRRs) and estimated adjusted hazard ratios (aHRs) with 95% confidence intervals (CIs) using Cox regression models, adjusting for age, sex, income levels, urbanization, cigarettes smoking, alcoholic related diseases, comorbidities, medication use, and Charlson Comorbidity Index (CCI) scores. The cumulative incidence of ESCC was estimated using the Kaplan–Meier method and compared using the log-rank test.

All statistical analyses were performed in SAS for Windows (version 9.4; SAS Institute, Cary, NC, USA), and a two-sided *p* < 0.05 was considered to indicate statistical significance.

## 3. Results

### 3.1. Baseline Characteristics of the Study Population

In this study, we analyzed data from 1,289,526 individuals who were enrolled between 2008 and 2018. The final follow-up date was 31 December 2020. To compare the AH user and nonuser groups, we performed individual 1:1 matching and each group included 644,763 patients. The age distribution was similar between the two groups (as shown in Table 1). After PSM, we found that the variables of sex, income levels, urbanization, cigarette smoking, alcoholic related diseases, comorbidities, medication use, and CCI scores were comparable between the AH user and nonuser groups, with no significant differences observed between the two groups.

### 3.2. Association of Comorbidities and Concurrent Medications with ESCC Risk

Table 2 presents the association of ESCC risk with concurrent medications and comorbidities in our study cohort. The risk of ESCC increased with age, with patients aged 18–50 years serving as the reference group. Men had a higher risk of ESCC than women, with an aHR of 2.31 (95% CI: 2.11–2.53). Cigarette smoking was associated with a higher risk of ESCC (aHR: 1.22; 95% CI: 1.13–1.66) and alcohol-related diseases was associated with an even higher risk (aHR: 2.14; 95% CI: 1.33–2.35) compared to non-smokers and those without alcohol-related diseases, respectively. However, the use of aspirin (aHR: 0.63; 95% CI: 0.56–0.71), metformin (aHR: 0.77; 95% CI: 0.66–0.90), and statins (aHR: 0.38; 95% CI: 0.33–0.44) was associated with a decreased risk of ESCC. Proton pump inhibitor (PPI) use was associated with an increased risk of ESCC (aHR: 1.22; 95% CI: 1.65–1.81).

### 3.3. IRs, IRRs, and aHRs for HCC among AH Users and Nonusers

Table 3 presents the relationship between AH use and ESCC development in our cohort. The incidence rate of ESCC was significantly higher in AH users than in nonusers (1.47 vs. 1.36 per 100,000 person-years). AH users had a higher incidence rate ratio for ESCC (95% CI) of 1.18 (1.08 to 1.28) compared to nonusers. After adjusting for age, sex, income levels, urbanization, cigarette smoking, alcohol-related diseases, comorbidities, medication use, and CCI scores, the risk of ESCC was significantly higher among AH users than nonusers (aHR: 1.22; 95% CI: 1.12 to 1.33).

**Figure 1:**
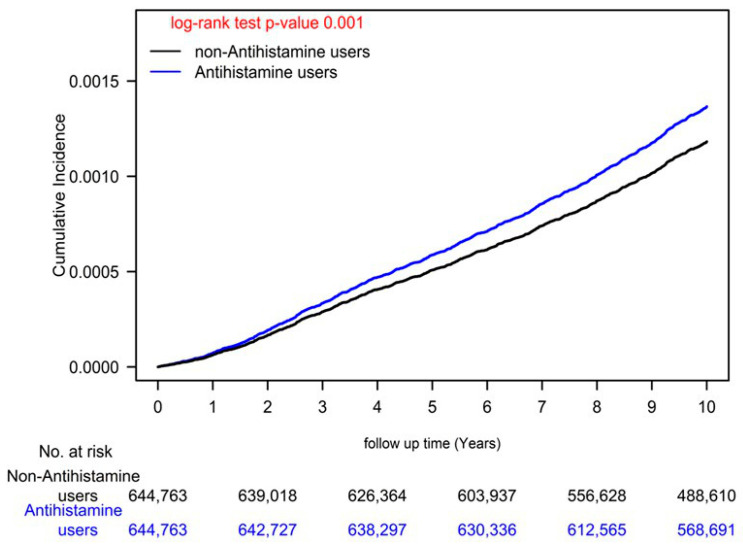
Cumulative incidence of ESCC relative to AH users and nonusers. AH, H1-antihistamine; ESCC, esophageal squamous cell carcinoma.

**Figure 2:**
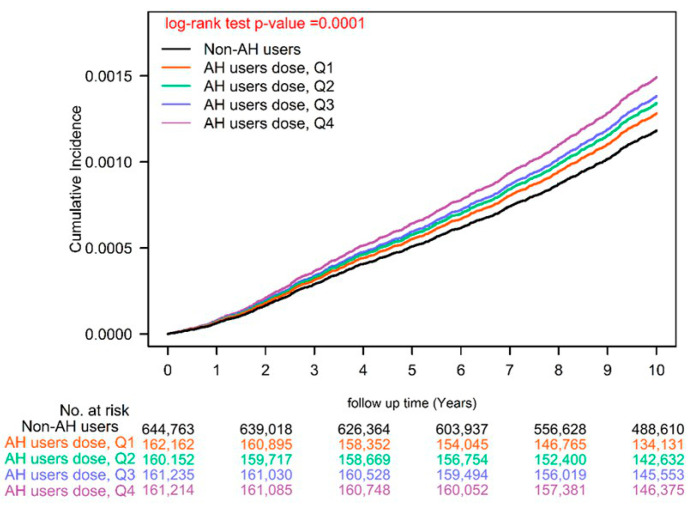
Kaplan–Meier cumulative incidence for ESCC at different cDDDs of AH use. AH, H1-antihistamine; ESCC, esophageal squamous cell carcinoma; cDDD, cumulative defined daily dose; Q, Quartile.

We also observed a dose–response relationship between AH use and ESCC risk: compared with AH nonuse (<28 cDDDs), the aHRs for AH use at 28–182, 183–488, 489–1043, and >1043 cDDDs were 1.12, 1.20, 1.25, and 1.37, respectively. Our Kaplan–Meier analysis showed that ESCC risk was higher in AH users than in AH nonusers (Figure 1; log-rank test, *p* = 0.001). Even after stratifying the patients by AH cDDDs, a similar trend was observed (Figure 2; log-rank test, *p* = 0.001).

### 3.4. Sensitivity Analysis

The results of the sensitivity analysis for age, sex, cigarettes smoking, alcohol-related diseases, DDD ≤ 1, DDD > 1, and medication use (aspirin, metformin, statin, PPIs) on the incidence of ESCC in individuals with and without AH use are shown in Table 4. The aHRs and 95% CIs for ESCC in AH users compared to nonusers showed a significantly higher incidence of ESCC in individuals aged 71 years or older, those who smoked cigarettes, those with alcohol-related diseases, and those with a daily dose of AH greater than or equal to 1.

## 4. Discussion

The results of our study were surprising, as we found a positive association between AH use and the incidence of ESCC, with a dose–response relationship between the two. This contradicts previous findings linking AH use to a decrease in the risk of HCC [13,15,36]. In our well-designed, large head-to-head comparative study using propensity score matching, we found that AH use was actually associated with an increased risk of ESCC (Table 3). The incidence rate of ESCC was significantly higher among AH users than non-users. Our study is the first to demonstrate a clear, dose-dependent relationship between AH use and the risk of ESCC using long-term follow-up and large data. This finding is in contrast to a previous study that found a non-significant trend towards an increased risk of esophageal cancer in AH users in subgroup analyses, with an odds ratio of 1.5 (95% CI: 0.9–2.5) [37]. However, that study had a small sample size and insufficient follow-up time, which may have contributed to the lack of statistical significance [37]. Our study adds to the evidence on the potential association between AH use and cancer risk, and highlights the need for further research on the effects of AH on different types of cancer and the possible underlying mechanisms.

H1-antihistamines are classified into two categories: first generation and second generation. First generation H1-antihistamines have poor selectivity for H1 receptors and can cross the blood–brain barrier. These drugs have been associated with a range of adverse events, including antimuscarinic, anti-alpha-adrenergic, anti-serotonin, and sedative effects. Despite these risks, the potential dangers of first-generation H1-antihistamines have been largely underestimated [11]. Until now, there has been no evidence linking the use of H1-antihistamines with the risk of ESCC. Our study is the first to examine the long-term use of H1-antihistamines and its association with ESCC risk. The reasons and mechanisms behind this association are currently unclear. One possibility is that the physiological side effects of H1-antihistamines, such as drowsiness [38] and lower esophageal sphincter pressure [37], may lead to abnormal gastrointestinal motility, resulting in inflammation of the esophagus and an increased risk of ESCC. It is also possible that AH may reduce the amount of gastric acid [39], similar to PPIs, which have been previously linked to an increased risk of esophageal cancer [40]. In our analysis with adjustments for age, sex, income levels, urbanization, cigarettes smoking, alcohol-related diseases, comorbidities, medication use, and CCI scores, AH use had an independent risk factor of ESCC development in our general population with dose–response relationship (Table 3, Figure 1 and Figure 2). According to our sensitivity analysis (see Table 4), the daily use of antihistamines (DDD > 1) was found to be an independent risk factor for ESCC with an adjusted hazard ratio (aHR; 95% CI) of 1.22 (1.10–1.34), regardless of other potential confounding factors. These findings suggest that more frequent, daily use of antihistamines may increase the risk of ESCC. Our study highlights the need for caution in the long-term use of H1-antihistamines as a safe anticancer medication, as the relationship between their use and cancer risk may vary depending on the type of cancer. Future clinical studies should re-evaluate the safety of long-term H1-antihistamine use in the general population.

Our study found that several factors are associated with an increased risk of ESCC, including older age (>50 years), the male sex, cigarette smoking, and alcohol-related diseases (see Table 2). These results are consistent with previous reports on the risk factors for ESCC [41,42,43,44]. In addition, we observed that the use of aspirin, metformin, and statins was associated with a reduced incidence of ESCC in our cohort (Table 2). These findings align with previous research suggesting that these medications may have a protective effect against ESCC [45,46,47]. However, the use of PPIs was associated with an increased risk of ESCC, consistent with the finding that PPIs may reduce the amount of gastric acid and potentially promote the development of esophageal cancer [40].

There are several strengths to this study, including its large sample size, large validation cohort, long-term follow-up time, homogenous covariates between cases and controls after PSM, and long-term verification of medication data. However, there are also some limitations to consider. First, although the National Health Insurance Administration routinely reviews patient charts to ensure the quality of claims from medical institutions, there is still the possibility of data miscoding or misclassification. Second, there are several unmeasured confounders related to ESCC (such as body mass index and use of other over-the-counter drugs) that were not included in our database. Third, the analysis of different H1-antihistamines is difficult because the crossover use of H1-antihistamines is very common in the AH users group. Unfortunately, it is difficult to separate the effects of different H1-antihistamines, as crossover use of these drugs is prevalent in the antihistamine user group. In our study, we faced the challenge of differentiating between individual use of specific H1-antihistamines. This is because, in practice, the choice of H1-antihistamines often depends on the availability and convenience of the drug at the hospital where it is prescribed. This leads to frequent crossover use of different H1-antihistamines. As a result, it is difficult to identify the exact antihistamine used by each individual, i.e., as crossover use is common. Fourth, we were unable to contact patients directly to confirm their AH use because their data were anonymized. We assumed that all patients adhered to their prescribed medication regimens, but the actual ingested dosage may have been overestimated due to nonadherence. One question that remains unanswered in this study is the duration of AH use. Our study showed that AH usage ≥28 cDDDs was a risk factor for ESCC. We also found that patients who were prescribed AH at a dosage of ≥28 cDDDs had a significantly increased risk of ESCC with a dose–response relationship (see Table 3 and Figure 2). However, in our sensitivity analysis examining the daily use of AH (DDD >1 or ≤ 1; see Table 4), we found that daily use (DDD > 1) was an independent risk factor for ESCC, suggesting that long-term, daily AH use may increase the risk of ESCC. Due to the low incidence of ESCC (see Table 1) in the general population, such an RCT would require a large sample size and may be difficult to perform. As an alternative, our long-term follow-up, head-to-head PSM comparative study may be a more suitable approach to addressing this issue. Finally, it should be noted that laboratory and clinical data were not readily available through the administrative database used in this study.

## 5. Conclusions

The use of AHs may increase the risk of ESCC in the general population in a dose-dependent manner. Further research is needed to confirm these findings and to understand the mechanisms behind this association. It is important for healthcare providers to consider the potential risks and benefits of AH use, especially in individuals at high risk for ESCC. Patients who are considering using AHs should discuss the potential risks and benefits with their healthcare providers.

## Figures and Tables

**Table 1:** Baseline Characteristics of AH User and Nonusers after PSM.

	AH Nonusers		AH Users		ASMD
	N = 644,763		N = 644,763		
	N	%	N	%	
**Age** (mean ± SD), years-old	54.86 ± 13.17		54.52 ± 13.06		0.001
Age, median (IQR, Q1, Q3), years-old	54.00 (43.00,66.00)		54.00 (43.00,67.00)		
Age group, years					0.000
≤50	286,941	44.50%	286,941	44.50%	
51–60	110,492	17.14%	110,492	17.14%	
61–70	101,162	15.69%	101,162	15.69%	
>70	146,168	22.67%	146,168	22.67%	
**Sex**					0.000
Female	308,603	47.86%	308,603	47.86%	
Male	336,160	52.14%	336,160	52.14%	
**Income (NTD)**					0.002
Low income	3458	0.54%	3631	0.56%	
≤10,000	304,981	47.30%	298,908	46.36%	
10,001–20,000	180,082	28.93%	177,490	27.53%	
20,001–30,000	67,170	10.42%	70,101	10.87%	
30,001–45,000	52,677	8.17%	57,260	8.88%	
>45,000	35,295	5.47%	37,373	5.80%	
**Urbanization**					0.002
Rural	153,647	23.83%	158,989	24.66%	
Urban	491,115	76.17%	485,772	75.34%	
**Cigarettes smoking**	8575	1.33%	8359	1.30%	0.001
**Alcoholic related diseases**	1418	0.22%	1321	0.20%	0.001
**Comorbidities**					
Diabetes	9993	1.55%	10,062	1.56%	0.001
Hypertension	21,844	3.39%	21,796	3.38%	0.001
Hyperlipidemia	8188	1.27%	8186	1.27%	0.000
Chronic Obstructive Pulmonary Disease	8314	1.29%	8359	1.30%	0.000
Gastroesophageal reflux disease	322	0.05%	429	0.07%	0.001
Barrett’s esophagus	293	0.05%	340	0.05%	0.000
Obesity	292	0.05%	343	0.05%	0.000
Achalasia	46	0.01%	47	0.01%	0.000
Tylosis (Howel-Evans syndrome)	320	0.05%	352	0.05%	0.000
Plummer–Vinson syndrome	22	0.00%	23	0.00%	0.000
**Medication Use**					
Aspirin	54,077	8.39%	91,324	14.16%	
Metformin	34,178	5.30%	50,840	7.89%	
Statin	47,681	7.40%	82,266	12.76%	
PPI	52,381	8.12%	95,553	14.82%	
**CCI Scores**					
Mean (SD)	0.09 ± 0.31		0.09 ± 0.42		0.001
Median (IQR, Q1-Q3)	0.00 (0.00,0.00)		0.00 (0.00,0.00)		
**CCI Scores**					0.001
0	611,236	94.80%	611,866	94.90%	
≥1	33,527	5.20%	32,897	5.10%	
**AH use**					
**cDDD**					
Nonuse	644,763	100.00%	0	0.00%	
Q1	0	0.00%	162,162	25.15%	
Q2	0	0.00%	160,152	24.84%	
Q3	0	0.00%	161,235	25.01%	
Q4	0	0.00%	161,214	25.00%	
**daily DDD**					
≤1	0	0.00%	468,111	72.60%	
>1	0	0.00%	176,652	27.40%	
					***p*** **Value**
Mean (+/- SD) follow-up, years	12.59 ± 3.66		13.85 ± 2.89		0.274
Median (IQR, Q1, Q3) follow-up, years	13.79 (10.15,15.55)		13.76 (12.62,15.93)		0.629
**Primary Outcome**					
**ESCC**					0.001
No	643,661	99.83%	643,542	99.81%	
Yes	1102	0.17%	1321	0.20%	

**Abbreviations:** AH, H1-antihistamine; cDDD, cumulative defined daily dose; IQR, interquartile range; SD, standard deviation; N, number; ASMD, absolute standardized mean difference; PSM, propensity scores matching; PPI, proton pump inhibitor; ESCC, esophageal squamous cell carcinoma; CCI, Charlson Comorbidity Index; DDD, defined daily dose; NTD, New Taiwan dollars.

**Table 2:** Association of Comorbidities and Concurrent Medications with ESCC Risk.

	Crude HR	(95% CI)	*p*	aHR *	(95% CI)	*p*
**AH use** (ref. nonuser)						
AH user	0.99	(0.91, 1.07)	0.725	1.22	(1.12, 1.33)	<0.001
**Sex** (ref. female)						
Male	2.04	(1.87, 2.23)	<0.001	2.31	(2.11, 2.53)	<0.001
**Age group**, years-old (ref. 18–50)						
51–60	1.47	(1.13, 1.95)	<0.001	1.45	(1.41, 1.88)	<0.001
61–70	1.76	(1.07, 1.91)	<0.001	1.52	(1.04, 1.77)	<0.001
>70	2.35	(1.44, 2.83)	<0.001	2.01	(1.20, 2.18)	<0.001
**Income** (Ref. Low income, NTD)						
≤10 000	1.03	(0.74, 1.76)	0.583	1.11	(0.88, 1.15)	0.516
10 001–20 000	1.36	(0.77, 2.4)	0.2915	1.16	(0.65, 2.04)	0.620
20 001–30 000	1.33	(0.75, 2.37)	0.327	1.04	(0.59, 1.86)	0.884
30,001–45 000	1.27	(0.71, 2.27)	0.414	0.88	(0.49, 1.57)	0.659
>45 000	1.01	(0.56, 1.81)	0.985	0.59	(0.33, 1.07)	0.082
**Urbanization** (Ref. rural)						
Urban	0.79	(0.72, 0.86)	<0.001	0.99	(0.9, 1.09)	0.833
**Cigarettes smoking** (Ref. non-smoker)	1.37	(1.16, 1.63)	0.0003	1.22	(1.13, 1.66)	0.020
**Alcoholic related diseases** (Ref. no Alcoholic related diseases)	2.19	(1.35, 2.91)	<0.001	2.14	(1.33, 2.35)	<0.01
**Comorbidities**						
Diabetes	4.29	(3.49, 5.27)	<0.001	1.90	(0.48, 2.43)	0.163
Hypertension	3.37	(2.87, 3.96)	<0.001	1.29	(0.87, 1.54)	0.257
Hyperlipidemia	2.02	(1.67, 2.45)	<0.001	1.80	(0.46, 2.22)	0.366
Chronic Obstructive Pulmonary Disease	2.41	(1.79, 3.25)	<0.001	0.85	(0.61, 1.16)	0.302
Gastroesophageal reflux disease	5.03	(1.26, 20.09)	0.0221	1.16	(0.76, 4.34)	0.433
Barrett’s esophagus	10.03	(4.78, 21.04)	<0.001	2.20	(0.73, 4.72)	0.442
Obesity	1.24	(0.18, 8.84)	0.8269	0.69	(0.1, 4.91)	0.710
Achalasia	11.43	(1.61, 80.91)	0.0147	8.98	(0.86, 14.13)	0.329
Tylosis (Howel-Evans syndrome)	1.19	(0.17, 8.32)	0.8633	0.44	(0.06, 3.16)	0.416
Plummer–Vinson syndrome	1.00	(0.43, 5.11)	0.9429	1.00	(0.66, 1.43)	0.918
**Medication Use**						
Aspirin	1.61	(1.44, 1.79)	<0.001	0.63	(0.56, 0.71)	<0.001
Metformin	1.46	(1.27, 1.67)	<0.001	0.77	(0.66, 0.9)	0.001
Statin	0.88	(0.77, 1.01)	0.0738	0.38	(0.33, 0.44)	<0.001
PPI	1.44	(1.29, 1.61)	<0.001	1.22	(1.65, 1.81)	<0.001
**CCI** (Ref. 0)						
CCI ≥ 1	2.95	(2.56, 3.4)	<0.001	1.23	(0.84, 1.46)	0.316

**Abbreviations:** aHR, adjusted hazard ratio; CI, confidence interval; AH, H1-antihistamine; HR, hazard ratio; CI, confidence interval; PPI, proton pump inhibitor; ESCC, esophageal squamous cell carcinoma; CCI, Charlson Comorbidity Index; NTD, New Taiwan dollars; Ref., reference group. *All covariates in Table 2 are adjusted.

**Table 3:** IRRs and aHRs for ESCC.

	Events	Person-Years	IR, 10, 000 Person-Year (per 100,000 Person-Year)	IRR	95%CI for IRR	aHR *	95%CI for HR	*p*
**AH use**								
Nonuse (≤28 cDDD)	1102	8,116,205.0	1.36	Ref.		Ref.		
>28	1221	8,930,408.0	1.47	1.18	(1.08, 1.28)	1.22	(1.12, 1.33)	<0.001
**AH use (cDDD)**								
Nonuse (<28 cDDD)	1102	8,116,205.0	1.36	Ref.		Ref.		
Q1, 182 cDDD	335	2,112,942.0	1.49	1.08	(0.95, 1.22)	1.12	(0.99, 1.27)	0.084
Q2, 488 cDDD	334	2,224,884.0	1.37	1.15	(1.01, 1.31)	1.20	(1.05, 1.36)	0.006
Q3, 1043 cDDD	318	2,291,045.0	1.28	1.20	(1.06, 1.37)	1.25	(1.1, 1.43)	0.001
Q4, 10,936 cDDD	334	2,301,536.0	1.34	1.32	(1.16, 1.5)	1.37	(1.2, 1.56)	<0.001

**Abbreviations**: aHR, adjusted hazard ratio; CI, confidence interval; AH, H1-antihistamine; HR, hazard ratio; CI, confidence interval; ESCC, esophageal squamous cell carcinoma; cDDD, cumulative defined daily dose; IQR, interquartile range; Q, Quartile; IR, incidence rate; IRR, incidence rate ratio; Ref., reference. * All covariates in Table 2 were adjusted.

**Table 4:** Sensitivity analyses of ESCC incidence–AH use association.

Subpopulation or Exposure	No. of Patients	ESCC Risk			
		No. of ESCC	aHR *	95% CI	*p*
**Age group, years**					
≤50	573,882	44	1.08	(0.36, 1.30)	0.246
51–60	220,984	307	1.10	(0.82, 1.31)	0.753
61–70	202,324	616	1.11	(0.94, 1.30)	0.219
≥71	292,336	1356	1.24	(1.11, 1.38)	<0.001
**Sex**					
Female	617,206	718	1.75	(1.50, 2.03)	<0.001
Male	672,320	1605	0.97	(0.87, 1.07)	0.498
**Cigarettes smoking**	16,934	3377	1·27	(1·05, 1·77)	<0.001
**Alcoholic related diseases**	2739	493	1·18	(1·07, 2·29)	<0.001
**DDD**					
≤1	986,911	1765	0.97	(0.81, 1.14)	0.685
>1	302,615	558	1.22	(1.10, 1.34)	<0.001
**Medication use**					
Aspirin	145,401	409	1.06	(0.86, 1.31)	0.571
Metformin	85,018	227	0.89	(0.68, 1.17)	0.402
Statin	129,947	228	1.15	(0.87, 1.52)	0.336
PPI	147,934	386	1.07	(0.63, 1.95)	0.214

**Abbreviations:** aHR, adjusted hazard ratio; CI, confidence interval; AH, H1-antihistamine; HR, hazard ratio; CI, confidence interval; ESCC, esophageal squamous cell carcinoma; DDD, defined daily dose; PPI, proton pump inhibitor.

## Data Availability

Data analyzed during the study were provided by a third party. Requests for data should be directed to the provider indicated in the Acknowledgments.

## Abbreviations

aHR: adjusted hazard ratio; CI, confidence interval; AH, H1-antihistamine; cDDD, cumulative defined daily dose; IQR, interquartile range; SD, standard deviation; N, number; SMD, standardized mean difference; HR, hazard ratio; CI, confidence interval; IR, incidence rate; IRR, incidence rate ratio; HCC, hepatocellular carcinoma; PSM, propensity scores matching; NHI, National Health Insurance; NHIRD, National Health Insurance Research Database; PPI, proton pump inhibitor; ESCC, esophageal squamous cell carcinoma; CCI, Charlson Comorbidity Index; DDD, defined daily dose.
